# SARS-CoV-2 Infects Endothelial Cells *In Vivo* and *In Vitro*

**DOI:** 10.3389/fcimb.2021.701278

**Published:** 2021-07-06

**Authors:** Fengming Liu, Kun Han, Robert Blair, Kornelia Kenst, Zhongnan Qin, Berin Upcin, Philipp Wörsdörfer, Cecily C. Midkiff, Joseph Mudd, Elizaveta Belyaeva, Nicholas S. Milligan, Tyler D. Rorison, Nicole Wagner, Jochen Bodem, Lars Dölken, Bertal H. Aktas, Richard S. Vander Heide, Xiao-Ming Yin, Jay K. Kolls, Chad J. Roy, Jay Rappaport, Süleyman Ergün, Xuebin Qin

**Affiliations:** ^1^ Division of Comparative Pathology, Tulane National Primate Research Center, Covington, LA, United States; ^2^ Department of Immunology and Microbiology, Tulane University School of Medicine, New Orleans, LA, United States; ^3^ Institute of Anatomy and Cell Biology, Julius-Maximilians-Universität Würzburg, Würzburg, Germany; ^4^ Department of Pathology and Laboratory Medicine, Tulane University School of Medicine, New Orleans, LA, United States; ^5^ Institute of Virology, Julius-Maximilians-Universität Würzburg, Würzburg, Germany; ^6^ Division of Hematology, Brigham and Women’s Hospital and Harvard Medical School, Boston, MA, United States; ^7^ Department of Pathology, LSU Health Sciences Center, New Orleans, LA, United States; ^8^ Departments of Medicine and Pediatrics, Center for Translational Research in Infection and Inflammation, Tulane University School of Medicine, New Orleans, LA, United States

**Keywords:** endothelial cell infection, animal models, SARS-CoV-2, aorta ring, hACE2

## Abstract

SARS-CoV-2 infection can cause fatal inflammatory lung pathology, including thrombosis and increased pulmonary vascular permeability leading to edema and hemorrhage. In addition to the lung, cytokine storm-induced inflammatory cascade also affects other organs. SARS-CoV-2 infection-related vascular inflammation is characterized by endotheliopathy in the lung and other organs. Whether SARS-CoV-2 causes endotheliopathy by directly infecting endothelial cells is not known and is the focus of the present study. We observed 1) the co-localization of SARS-CoV-2 with the endothelial cell marker CD31 in the lungs of SARS-CoV-2-infected mice expressing hACE2 in the lung by intranasal delivery of adenovirus 5-hACE2 (Ad5-hACE2 mice) and non-human primates at both the protein and RNA levels, and 2) SARS-CoV-2 proteins in endothelial cells by immunogold labeling and electron microscopic analysis. We also detected the co-localization of SARS-CoV-2 with CD31 in autopsied lung tissue obtained from patients who died from severe COVID-19. Comparative analysis of RNA sequencing data of the lungs of infected Ad5-hACE2 and Ad5-empty (control) mice revealed upregulated KRAS signaling pathway, a well-known pathway for cellular activation and dysfunction. Further, we showed that SARS-CoV-2 directly infects mature mouse aortic endothelial cells (AoECs) that were activated by performing an aortic sprouting assay prior to exposure to SARS-CoV-2. This was demonstrated by co-localization of SARS-CoV-2 and CD34 by immunostaining and detection of viral particles in electron microscopic studies. Moreover, the activated AoECs became positive for ACE-2 but not quiescent AoECs. Together, our results indicate that in addition to pneumocytes, SARS-CoV-2 also directly infects mature vascular endothelial cells *in vivo* and *ex vivo*, which may contribute to cardiovascular complications in SARS-CoV-2 infection, including multipleorgan failure.

## Introduction

The confirmed case count of the global COVID-19 pandemic caused by severe acute respiratory syndrome coronavirus 2 (SARS-CoV-2) is now in excess of a hundred million, with over two million deaths. SARS-CoV-2, a single-stranded RNA virus, probably crossed from bats to humans following a gain-of-function mutation in the spike (S) protein (Zou et al., 2020). Angiotensin-converting enzyme 2 (ACE2) plays a crucial role in SARS-CoV-2 infection and is believed to serve as the major entry receptor for the virus in humans (Datta et al., 2020). The FDA has authorized several vaccines targeting the S protein for emergency use to help control the current pandemic. Although rapid worldwide vaccination may curtail the spread of SARS-CoV-2, there is an urgent need to better understand its pathobiology for two important reasons. First, SARS-CoV-2 has shown the ability to sustain critical mutations that increase its infectivity, so it is likely that the virus in circulation will continue to mutate and spread, especially in parts of the world where vaccines will not be widely available. It is possible that some of these mutations will render current vaccines less effective, thus starting a new arms race between the virus and host. The new variant B.1.1.7, in the UK has 17 amino acid mutations, eight of which are in the S gene, and appears to be better at spreading between people. The new variant B.1.351 is driving a resurgence of cases in South Africa and has raised concerns about the efficacy of current vaccines. Second, given the emergence of three deadly coronavirus infections (SARS, MERS, COVID-19) from 2003 to 2019, it is almost inevitable that new coronavirus pandemics will continue to emerge. There are thousands of beta-coronaviruses in bats and perhaps other hosts with pandemic potential (Woo et al., 2012; Totura and Bavari, 2019). Therefore, a better understanding of the pathobiology of SARS-CoV-2 is essential for developing novel host- and/or virus-directed treatment strategies.

The typical clinical manifestations of COVID-19, SARS, and MERS include fever, cough, and shortness of breath that can progress to pneumonia, which in turn activates immune cells, platelets, and coagulation pathways, leading to a massive cytokine release (cytokine storm), and ultimately multiple organ failure and death (Datta et al., 2020). Mounting clinical and experimental evidence demonstrates that SARS-CoV-2 infection induces immune dysfunction, widespread endothelial injury, coagulopathy and systemic microangiopathy (Perico et al., 2021). Increased vascular permeability due to infection and inflammatory response plays a critical role in disease outcome, as it is the leakage of cells and plasma components from the pulmonary small blood vessels that causes alveoli to fill with liquid, resulting in asphyxiation. Clinical studies using serum biomarker analysis and histological analyses, including electron microscopy (EM) studies, indicate that the virus may also infect endothelial cells in a variety of tissues, especially in the lung (Fox et al., 2020; Goshua et al., 2020; O’Sullivan et al., 2020; Varga et al., 2020a). The identification of SARS-CoV-2 in endothelial cells from infected human samples by EM has led to a debate over whether these cells are infected by the virus (Goldsmith and Miller, 2020; Roufosse et al., 2020). Engineered human capillary organoids, generated from induced pluripotent stem cells were successfully infected with SARS-CoV-2, as confirmed by recovery of viral RNA post-infection (Monteil et al., 2020). However, recent studies show that primary human ECs lack ACE2 expression at the protein and RNA levels and that SARS-CoV-2 is incapable of directly infecting primary ECs (Nascimento Conde et al., 2020; McCracken et al., 2021). Some *in vitro* culture studies suggest that endothelial cells are resistant to SARS-CoV-2 infection (Blerina Ahmetaj-Shala et al., 2021) and only alveolar epithelial cells are infected by SARS-CoV-2 (Wang et al., 2020). Even as clinical reports of COVID-19-related vasculopathy grow, there has been little evidence supporting direct infection of the endothelium by SARS-CoV-2 *in vitro*. The failure to recapitulate these clinical observations in the laboratory could be due to differences between the endothelial monolayer culture commonly used in the laboratory and the endothelial layer lining the blood vessels under sheer stress, endothelial activation by SARS-CoV-2-infection-related cytokine storm, or in the case of pulmonary capillaries, very tight contact with pulmonary epithelial cells (Wazny et al., 2020). Understanding whether SARS-CoV-2 infects the endothelial cells is of critical importance, because if the virus can breach the endothelial barrier, it will have the opportunity to infect smooth muscle cells in medium and large blood vessels and cardiomyocytes in the heart, all of which express ACE2. We therefore addressed the question of whether SARS-CoV-2 can infect endothelial cells after lung epithelial infection using a hACE2-expressing mouse model of COVID-19 (Han et al., 2021), archived lungs from SARS-CoV-2-infected nonhuman primates, and autopsied lungs from COVID-19 patients. We also used an organ culture model of mouse aortic ring assay (ARA) first introduced by Nicosia and Ottinetti (1990) to investigate endothelial cells infection *in vitro*.

We found that both SARS-CoV-2 proteins and viral RNA co-localize with the endothelial cell marker CD31 using immunofluorescence and RNAscope, respectively. We also detected SARS-CoV-2 particles in endothelial cells of lung vessels *via* immune electron microscopic analysis in the lung tissue sections of infected Ad5-hACE2 mice. We performed immunohistochemical and electron microscopic studies on mouse aortic tissue sections obtained from small aortic rings (ARs) after being used in ARA for 6 days to induce an endothelial activation followed by exposure to SARS-CoV-2 for additional 3 days, and demonstrated that SARS-CoV-2 efficiently infects aortic endothelial cells (AoECs). Moreover, double immunostaining for CD34 and SARS-CoV-2, CD34 and ACE2, as well as SARS-CoV-2 and ACE2 revealed that compared to tissue sections of quiescent mouse aorta, endothelial cells in tissue sections of infected mouse aortic rings were positive for ACE2, suggesting an upregulation of ACE2 in endothelial cells upon activation. This endothelial activation by ARA may resemble the endotheliopathy caused by SARS-CoV-2 infection. ARA using mouse aorta, or even pieces of human artery, seems to be a suitable *ex vivo* organ model for studying infection-related vascular pathology and drug efficacy. Taken together, our results indicate that SARS-CoV-2 also infects endothelial cells *in vivo* and *ex vivo*, which likely contributes to the pathogenesis of COVID-19 including cardiovascular complications and multiple organ failure.

## Materials and Methods

### Mice and Ethics Statement

Wild-type C57BL/6J mice of 6- to 12-weeks were housed and bred in the animal facility of Tulane University School of Medicine. The Institutional Animal Care and Use Committee of Tulane University reviewed and approved all procedures for this experiment (permit number P0443). The Tulane National Primate Research Center (TNPRC) is fully accredited by the Association for Assessment and Accreditation of Laboratory Animal Care (AAALAC).

### SARS-CoV-2 Infection

Mice were oropharyngeally transduced with 1.5 x 10^9^ plaque-forming unit (pfu) of Ad5-hACE2 or Ad5-empty vectors (Vector Biosystems Inc, Malvern, PA) under Animal Biological Safety Level 2 (ABSL2) conditions. Four days post-transduction, mice were intranasally infected by SARS-CoV-2 in an ABSL3 lab with 5×10^4^ median tissue culture infectious dose (TCID_50_) per nostril.

### SARS-CoV-2 Infection of Mouse Aortic Ring Assays

Aortic ring assays were performed by first dissecting and cutting mouse aortas into rings of approx. 1 mm and keeping them at serum starvation overnight. The following day, the thoracic aortic rings were embedded with, the lumen facing the top, in collagen gel (collagen type I, rat tail, Millipore, Cat# 08-115, 1 mg/ml, PH=7 adjusted *via* 5N NaOH), using a 96- well plate. They were cultivated in Opti- MEM (Thermo Fischer sci., 51985-026) + 2, 5% FCS (heat-inactivated, Biochrom, S0115) + 30 ng/ml VEGF-a (Peprotech, 450-32). On day 6, SARS-CoV-2 was added (5 X 10^5^ infectious viruses) to their medium at an ABSL3 lab for 3 more days. The aortic rings were washed twice with PBS and fixed with either 4% PFA, Roti-Histofix (Roth, Germany) or with fixation solution (0.12M PB buffer pH 7.4 containing 1% glutaraldehyde, 1% formaldehyde with 2mM calcium chloride and 2% sucrose).

### Histological Analysis and Scoring of Perivascular Inflammation

Lung sections were processed routinely, stained with hematoxylin and eosin (H&E), digitally scanned by a Zeiss Axio Scan.Z1 creating whole-slide images, and analyzed by a board-certified pathologist with computer software (HALO v3.1, Indica Labs) using two algorithms (Multiplex IHC v2.3.4 and Spatial Analysis). Annotation regions were drawn around small arterioles, then all nucleated cells were counted using Multiplex IHC v2.3.4. Spatial Analysis was used to quantify the number of nucleated cells within a 100μm radius of the annotated vessels. Perivascular inflammation was reported as the density of nucleated cells within a 100μm radius of the tunica adventitia of small arterioles (nucleated cells/mm^2^).

### Immunohistochemistry

After deparaffinization, formalin-fixed, paraffin-embedded (FFPE) lung sections were heated in a high-pH solution (Vector Labs H-3301), rinsed in hot water and transferred to a heated low-pH solution (Vector Labs H-3300) until cooled to room temperature (RT) for antigen retrieval. Sections were blocked with 10% normal goat serum (NGS) and incubated with the primary antibodies for 60 minutes and secondary antibodies for 40 minutes at RT. Following washes, 4`,6-diamidino-2-phenylindole (DAPI) was used to label the nuclei. Slides were mounted using a homemade anti-quenching mounting media containing Mowiol (Calbiochem, Cat# 475904) and DABCO (Sigma, Cat# D2522).

Fluorescently labeled slides were digitally scanned by a Zeiss Axio Scan.Z1 to generate whole-slide images that were quantified using computer software (Highplex FL, HALO, Indica labs) to recognize cells and quantify fluorescence in each channel (405nm, 488nm, 568nm, and 647nm). Thresholds of positivity for each marker/channel were set by board-certified pathologists. Regions of interest were drawn around each piece of lung on a slide, and all annotated regions were analyzed using the thresholds established by the pathologist for that staining panel. The accuracy of the analysis was visually confirmed by the pathologist. For each panel, the number of SARS-CoV-2 positive cells with/without a co-expressed phenotypic marker was summated for all mice (n=4 to 5).

### Immunohistochemistry/Immunofluorescence of ARA Sections

Aortic ring assays were fixed in 4% PFA solution for 48-72h, removing the SARS-CoV-2 containing supernatant beforehand, washed in PBS, embedded in paraffin and sectioned (10µm) for further analyses. Sections were deparaffinized and antigens were unmasked using sodium citrate buffer (10mM, pH6). Blocking steps *via* endogenous peroxidase block (3% H2O2 in demineralized water, 10 min, RT) and blocking solution (4% BSA, 0,2% Triton X- 100 in PBS, 2h, RT) followed. Immunoreaction was developed by applying either DAB procedure alone or a combination of DAB and immunofluorescence staining. For DAB staining, peroxidase and avidin biotin complex methods were applied, and SARS-CoV-2 was detected *via* guinea pig anti-SARS-COV-2 polyclonal antiserum (BEI Resources, Cat# NR-10361). VCAM-1 was detected *via* rabbit anti- VCAM-1 (Santa Cruz, SC-1504). For immunofluorescence, rat anti-CD34 (Abcam, #ab8158), rabbit anti-CD31 (Abcam, #ab 28364) and goat anti-ACE2 (R&D systems, AF933) were applied as primary antibodies. Secondary Cy3-, Cy5- and Alexa Fluor 647-labeled as well as biotinylated antibodies were used to visualize primary antibodies. For labeling the nuclei, either 0.1% nuclear fast red solution (in DAB only) or DAPI were used. Specimens were analyzed with a Keyence Biozero 9000 microscope. SARS-CoV-2-infected human nasal mucosa was used as positive control, while non-infected aortic ring assays served as negative controls.

### PCR and Quantitative Real-Time PCR of ARA and FIA

PCR and Quantitative Real‐Time PCR of ARA and FIA: after fresh RNA was isolated aorta in aortic ring assay (n=2), each sample mRNA expression was analyzed by real- time PCR in duplicate or triplicate (GoTaq qPCR Master Mix, Promega). Primers used in this study were GAPDH (Mm GAPDH UP1CCA GGT TGT CTC CTG CGA CT, Mm GAPDH DP1 ATA CCA GGA AAT GAG CTT GAC AAA G) and VCAM1 (Mm VCAM1 UP1 AAA CGC GAA GGT GAG GAC GG, Mm VCAM1 DP1 CAC TTG ACC GTG ACC GGC TT). After completion of the qPCR cycling protocol (95°C for 30 s; 60°C for 30 s; 72°C for 30 s; 50×), specificity of amplification was proven by melting curve analyses. For PCR the same primers and cycling protocol were used, 35 cycles. Instead of Go qPCR Master Mix, Red Taq Master Mix (Genaxxon bioscience) was used (n=2).

### RNAscope

All procedures followed the protocol from Advanced Cell Diagnostics (ACD). FFPE lung slides were deparaffinized in fresh xylene and fresh 100% ethanol and then air-dried. Target retrieval was performed (RNAscope^®^ Target Retrieval Reagents, ACD Cat# 322000) after hydrogen peroxide treatment and was followed by protease treatment (RNAscope^®^ H2O2 & Protease Plus, ACD Cat# 322330). Probes for RNAs of platelet endothelial cell adhesion molecule (*Pcam1*, also known as *CD31)* (RNAscope^®^ Probe *Mm-pcam1*, ACD Cat# 316721) and *spike* protein (RNAscope^®^ Probe *V-nCoV2019-S-C2*, ACD Cat# 848561-C2) were mixed and incubated on slides for *in situ* hybridization. Following signal detection (RNAscope^®^ 2.5 HD Duplex Detection Reagents, ACD Cat# 322500), slides were stained with 50% hematoxylin (Hematoxiylin Solution, Gill No.1, Sigma-Aldrich, Cat# GHS132) and mounted with VectaMount Permanent Mounting Media (ACD, Cat# 321584).

### Electron Microscopy of ARA

#### Standard Electron Microscopic Preparation

Specimens were fixed in fixation solution (0.12M PB buffer pH 7.4 containing 1% glutaraldehyde, 1% formaldehyde with 2mM calcium chloride and 2% sucrose) on ice for 1 hour and washed 5 x 3 minutes in cold 0.15M cacodylate buffer (50mM cacodylate, 50mM KCl, 2.5mM MgCl_2_, 2mM CaCl_2_ pH 7.4) on ice. After washing for 4 x 5 minutes in 0.15M cacodylate buffer at RT, specimens were subsequently fixed for 1 hr with 1% osmium tetroxide in 0.15M cacodylate buffer. Specimens were washed 2 x 5 minutes in ddH_2_O. Afterwards, specimens were incubated for 1 hr in aqueous UAR-EMS (4%, Uranyl Acetate Replacement Stain, Electron Microscopy Sciences, Hatfield, USA), washed 2 x 5 minutes in ddH_2_O at RT and dehydrated in an ascending ethanol series using solutions of 30%, 50%, 70%, 90%, 96%, and 100% ethanol for 10 minutes each. They were incubated two times in propylene oxide (PO) for 30 minutes each before incubation in a mixture of PO and Epon812 (1:1) overnight. The following day, the Epon-PO mixture was substituted with pure Epon812 and samples were incubated for 2 hours in Epon812. Specimens were embedded in Epon812 and kept at 60°C for 48hrs.

#### High Contrast Electron Microscopic Preparation

To enhance membrane contrast, specimens were fixed in fixation solution (0.12M PB buffer pH 7.4 containing 1% glutaraldehyde, 1% formaldehyde with 2mM calcium chloride and 2% sucrose) on ice for 1 hour and washed 5 x 3 minutes in cold 0.15M cacodylate buffer (50mM cacodylate, 50mM KCl, 2.5mM MgCl_2_, 2mM CaCl_2_ pH 7.4) on ice. Subsequently, specimens were incubated for 1 hr on ice in a reduced osmium solution containing 2% osmium tetroxide, 1.5% potassium ferrocyanide, 2 mM CaCl_2_ in 0.15 mM sodium cacodylate buffer (pH 7.4). Specimens were washed with ddH_2_O at room temperature (RT) 5 x 5 minutes, followed by incubation in 1% thiocarbohydrazide (TCH) solution for 25 minutes at RT. Specimens were washed with ddH_2_O at RT, 5 x 5 minutes each and incubated in 2% osmium tetroxide in ddH_2_0 for 30 minutes at RT. Afterwards, specimens were incubated in aqueous UAR-EMS (4%, Uranyl Acetate Replacement Stain, Electron Microscopy Sciences, Hatfield, USA) and stored at 4°C overnight. The next day, specimens were washed 3 x 3 minutes in ddH_2_O at RT. Prior to incubation with lead aspartate solution, specimens were washed 2 x 3 minutes in ddH_2_O at 60°C and subjected to *en bloc* Walton’s lead aspartate staining (Walton, 1979) and placed in a 60°C oven for 30 minutes. Specimens were washed 5 x 5 minutes with ddH_2_O at RT and dehydrated using ice-cold solutions of freshly prepared 30%, 50%, 70%, 90%, 100%, and 100% ethanol (anhydrous), 100% acetone (anhydrous) for 10 minutes each, then placed in ice-cold anhydrous acetone and left at RT for 10 minutes. Specimens were placed in 100% acetone at RT for 10 minutes. During this time, Epon812 was prepared. The resin was mixed thoroughly, and samples were placed into 25% Epon:acetone for 2 hours, then into 50% Epon:acetone for 2 hours and 75% Epon:acetone for 2 hours. Specimens were placed in 100% Epon overnight. The next day, Epon was replaced with fresh Epon for 2 hours and specimens were placed in beem capsules and incubated in a 60°C oven for 48 hours for resin polymerization.

For ultrathin sections, 70nm thick ultrathin sections were cut with an ultramicrotome (Ultracut E, Reichert Jung, Germany) and collected on copper or nickel grids and finally analyzed with a LEO AB 912 transmission electron microscope (Carl Zeiss Microscopy GmbH, Germany).

### Post-Embedding Immunogold Electron Microscopy

Lung tissue cubes were fixed in zinc-formalin (Z-Fix) buffer, then dehydrated and embedded with Lowicryl Monostep K4M Embedding Media (Electron Microscope Sciences (EMS), Cat# 14335) at -35°C under ultraviolet (UV) rays for 72h. Ultrathin (100nm) sections were stained with anti-SARS coronavirus polyclonal antiserum (1:100, BEI Resources, Cat# NR-10361) at 4°C overnight. After washing with PBS, secondary goat anti-guinea pig IgG 6nm (1:20, EMS, Cat# 25324) was incubated for 1h at room temperature. Images were taken under FEI Tecnai G2 F30 Twin Transmission Electron Microscope.

### Over-Enrichment Analysis

Enrichment analysis was performed with the R package ClusterProfiler at default parameters. Enrichment scores were calculated against the following 4 pathway databases containing a priori-defined gene sets: Gene Ontology (GO) database, Hallmark (H) and Curated (C2) gene sets of the Molecular Signature database (MsigDB), and WikiPathways. Gene sets significantly enriched in the datasets (p < 0.05) were subsequently curated for those relevant to endothelial cell biological function. Enrichment plot was generated with the R software package ‘ggplot2’ and heatmaps with the ComplexHeatmap package.

## Results

### SARS-CoV-2 Infects Endothelial Cells in a Human ACE2 (hACE2) Mouse Model

**Figure 1:**
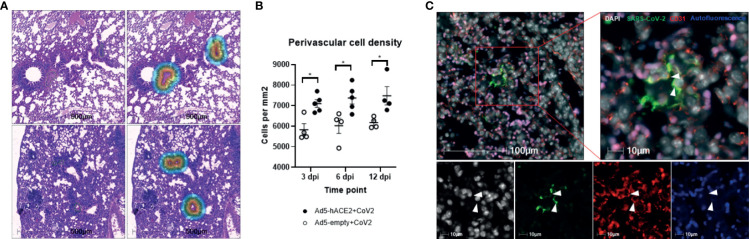
Perivascular inflammation and infection of pulmonary ECs by SARS-CoV-2 in Ad5-hACE2 mice. **(A)** Perivascular inflammation in both Ad5-empty (top images) and Ad5-hACE2 mice (bottom images) 3 days post infection (dpi). Control Ad5-empty mice have lower numbers of nucleated cells compared to Ad5-hACE2 mice. The mask of the infiltration analysis (top right and bottom right) shows the 100μm interface surrounding the tunica adventitia where the cell density was quantified: 5,461 cells/μm (top right) and 8,775 cells/μm (bottom right). **(B)** Quantification of perivascular inflammation of SARS-CoV-2 (CoV-2) infected mouse lungs on 3, 6, and 12 dpi. Perivascular inflammation was quantified by the density of nucleated cells within 100μm of the tunica adventitia of small arterioles, n = 4-6 for each time point per group. * indicates P<0.05 using two-tailed, unpaired t-test. **(C)** Representative immunostaining images showing co-localization (arrowhead) of SARS-CoV-2 proteins with CD31 in the lung of infected Ad5-hACE2 mice on 3 dpi. Autofluorescence (blue) differentiates red blood cells which fluoresce in the 488 (green), 568 (red), and 647 (blue) channels. Green: SARS-CoV-2; white: DAPI; red: CD31; blue: autofluorescence.

**Figure 2:**
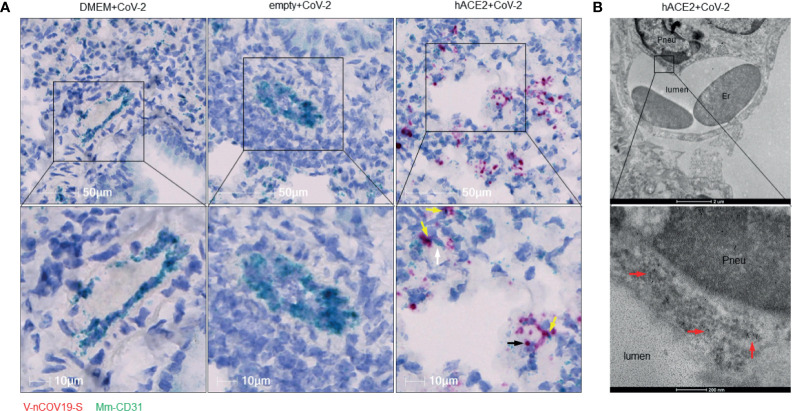
RNA of SARS-CoV-2 spike protein can be found inside endothelial cells. **(A)** Representative RNAscope images showed co-localization of RNA of spike protein with *CD31* RNA in SARS-CoV-2 infected Ad5-hACE2 mouse lungs (right panel, n = 2), but not in lungs of control mice transduced by DMEM (left panel, n = 3) or Ad5-empty (middle panel, n = 3). White arrow: Spike RNA signals showing light green; black arrow: *CD31* RNA signals showing red; yellow arrows: co-localization of two RNAs showing purple. **(B)** Immunoelectron microscope showed SARS-CoV-2 proteins (black particles indicated by red arrows, n = 1) inside endothelial cells of infected Ad5-hACE2 mice lungs. Pneu, pneumocyte; Er, erythrocyte.

Previously, we reported a rapidly deployable COVID-19 mouse model (Han et al., 2021). We expressed hACE2, an entry receptor for SARS-CoV-2 infection, in the lung by oropharyngeal transduction with a recombinant human adenovirus type 5 that expresses hACE2 (Ad5-hACE2) (Han et al., 2021). At day 4 post-transduction, Ad5-hACE2 mice were infected with SARS-CoV-2. The mice developed mild to moderate COVID-19 lung histology changes showing interstitial pneumonia and pulmonary perivascular inflammation with significantly higher viral loads in lungs at days 3, 6, and 12 post-infection compared to the Ad5-empty control group (Han et al., 2021). Consistent with the current view that SARS-CoV-2 mainly targets pneumocytes, we demonstrated that SARS-CoV-2 was detected in pneumocytes lining the surface of lung alveoli (Han et al., 2021). To investigate whether SARS-CoV-2 also infects vascular endothelial cells *in vivo*, we further characterized these COVID-19 mice (Han et al., 2021) by quantitatively analyzing pulmonary perivascular inflammation and endothelial cell infection. We found that SARS-CoV-2-infected Ad5-hACE2 mice had greater levels of pulmonary vascular inflammation than the Ad5-empty mice, as evidenced by a significantly higher number of nucleated cells that infiltrated into the pulmonary tissue with a particular accumulation within and around the adventitial layer of venules and arterioles from day 3 to day 12 post-infection (**Figures 1A, B**). These findings are consistent with previously published results from clinical studies and nonhuman primates showing that SARS-CoV-2 not only causes interstitial pneumonia but also pulmonary vascular inflammation (Han et al., 2021).We further explored the mechanism underlying SARS-CoV-2 infection-mediated vascular inflammation. We used immunofluorescence, RNAscope, and immune electron microscopic studies to detect the presence of SARS-CoV-2 in pulmonary endothelial cells of SARS-CoV-2-infected Ad-hACE2 mice at 3 days post-infection (DPI), when the viral load in the lung is at its highest (Han et al., 2021). We showed that SARS-CoV-2 is co-localized with CD31, a cell surface marker for endothelial cells that line the lumen of all blood vessels including those localized within the lung alveolar septa (**Figure 1C**), indicating that SARS-CoV-2 infects endothelial cells. As expected, we also observed that SARS-CoV-2 co-localizes with pan-cytokeratin (Pan-CK)-positive pneumocytes of the lung alveoli (**Supplemental Figure 1**). With RNAscope analysis, both large blood vessels and capillaries can be labeled by a probe that detects CD31. However, co-localization of CD31 with SARS-CoV-2 spike RNA could only be detected in capillaries of infected Ad5-hACE2 mouse lungs (**Figure 2A**), suggesting endothelial infection mostly occurs in the pneumocyte-endothelial barrier where gas exchange takes place, and pneumocytes and endothelial cells are separated from each other by only a thin basal lamina. Furthermore, using immune electron microscopy, we also observed SARS-CoV-2 virus particles in the endothelial cells of pulmonary capillaries, which only hold one or two erythrocytes (**Figure 2B**). Together, these results demonstrate that SARS-CoV-2 can infect endothelial cells in addition to pneumocytes in this model. Consistently, RNA sequencing from the lungs of Ad5-hACE2 vs Ad5-empty mice at 3 DPI revealed that interferon (Ifn)-gamma and alpha, allograft rejection, complement and KRAS signaling are upregulated in response to SARS-CoV-2-infection (**Supplemental Figure 2A**). Interestingly, KRAS signaling components that are upregulated in SARS-CoV-2 infected mice (**Supplemental Figure 2B**), such as complement factor B (cfb), Sperpina and GPNMB and CTSS, mediate activity of various pro-inflammatory molecules such as TNF-α and are involved in endothelial activation and angiogenesis (Hu et al., 2013; Salazar-Olivo et al., 2014; Rathi et al., 2017; Ni et al., 2020). These molecules may also contribute to endothelial activation and migration, which results in the increased vascular permeability that is the hallmark of fluid leakage and extravasation of inflammatory cells in the lungs of COVID-19 patients.

### Detection of SARS-CoV-2 in Pulmonary Endothelial Cells of Infected Non-Human Primates (NHPs) and Patients Who Died of COVID-19

**Figure 3:**
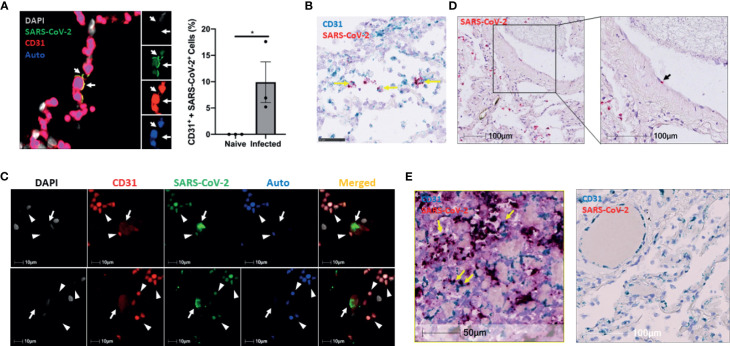
The infection of pulmonary ECs by SARS-CoV-2 in SARS-CoV-2 infected non-human primates and patients who died from severe COVID-19. **(A)** Representative image (left panel, n = 3) shows colocalization (yellow, arrows) of SARS-CoV-2 (green) and CD31 (red) in the lung of an African green monkey (AGM). Channel separation (right in inset) demonstrates that the SARS-CoV-2 and CD31 signal form an identical pattern, and that there is no autofluorescence (blue) in these areas. Quantitative analysis of SARS-CoV-2-infected endothelial cells in the lung of Naïve and infected AGM (right panel). Multilabel immunofluorescence histochemistry was used to quantify the proportion of SARS-CoV-2 positive CD31+ endothelial cells in total infected cells in the lung of AGMs (PA16, PA20 and PA24). White=DAPI, green=SARS-CoV-2, red=CD31, and blue=autofluorescence. *P < 0.05 vs naïve monkeys by one tail student T test **(B)** Representative RNAscope image of SARS-CoV-2 and CD31 RNA in the lung of infected AGM (n = 3). Yellow arrows: co-localization of Spike (shown in red) with CD31 (shown in light blue) RNA signal (shown in purple). **(C)** Colocalization of SARS-CoV-2 with CD31 in two cells (arrows) of a patient that died from COVID-19 (Case 3). Channel separation distinguishes true signal from red blood cells (arrowheads) that lack nuclei (DAPI channel) and can be seen in the autofluorescent/empty channel (blue). **(D)** RNAscope of SARS-CoV-2 RNA (red, black arrow) in the lung of the decreased COVID-19 patient (Case 3) **(E)** RNAscope of SARS-CoV-2 RNA (red) and CD31 RNA (green) in the lung of Case 2 and Case 1. Co-localization of SARS-CoV-2 RNA with CD31 was found in the lung of Case 2 patient (left panel) but not Case 1 patient (right panel). Yellow arrows: co-localization of *Spike* (showing in red) with CD31 (shown in light blue) RNA signal (shown in purple).

We further tested our findings using a non-human primate model, which very closely recapitulates human physiology and COVID-19 pathology. To this end, we used three archived lungs obtained from SARS-CoV-2-infected African green monkeys (AGMs) euthanized early in the course of the disease (4-6 DPI) (**Supplemental Table 1**). SARS-CoV-2 infection in AGMs can cause a wide range of disease from mild to severe COVID-19 (Blair et al., 2020; Chandrashekar et al., 2020; Cross et al., 2020; Hartman et al., 2020; Munoz-Fontela et al., 2020). We stained the lung sections for SARS-CoV-2 and CD31 (endothelial cell marker) to investigate endothelial cell infection and the co-localization of SARS-CoV-2 with CD31 (**Figure 3A**). Interestingly, we also detected the virus in CD3+ T cells, but only slightly in IBA-1+ macrophages (**Supplemental Figures 3A–C**). Furthermore, RNAscope data confirmed the presence of viral RNA in CD31 positive cells, validating our IHC results that SARS-CoV-2 infects endothelial cells in non-human primates (**Figure 3B**). To further determine the relevance of endothelial cell infection to the pathobiology of COVID-19, we examined lung autopsy samples from two adult COVID-19 patients (Case 1 and Case 3) who died from severe COVID-19 and one decreased neonate (Case 2) whose mother was positive for SARS-CoV-2 (**Supplemental Material**). The lung histological changes of three patients include acute and chronic inflammatory cells in the intra-alveolar space, intra-alveolar fibrin deposition, and diffuse hemorrhages (**Supplemental Figure 4**). Of note, Case 2 is a male neonate delivered prematurely by Cesarean section at 25.6 weeks gestational age due to pregnancy complications including chronic hypertension and severe preeclampsia. The mother tested positive for SARS-CoV-2 on the date of admission upon routine screening, although she was clinically asymptomatic. While we did not detect any virus in the lung of Case 1, we observed extensive SARS-CoV-2 proteins and N protein RNA by immunostaining and RNAscope in the lungs of Cases 2 and 3 (**Supplemental Figures 5A, B**). We found that SARS-CoV-2 co-stained with CD31 in some endothelial cells of interalveolar capillaries in Case 3, providing additional evidence that SARS-CoV-2 infects endothelial cells (**Figure 3C**). Of note, the capillaries were detached from the alveolar space and had a swollen appearance (**Figure 3C**); however, it is unclear whether this was a result of severe lung injury caused by infection or an artifact of postmortem autolysis. Using RNAscope, we also observed viral RNA in vascular endothelial cells in the pulmonary vessels of the Case 3 patient (**Figure 3D**). Moreover, we detected extensive viral RNA, some of which co-localized with CD31+ endothelial cells, in Case 2 (**Figure 3E**, left panel), but no virus was present Case 1 (**Figure 3E**, right panel). Together, our results indicate that endothelial cells are indeed susceptible to SARS-CoV-2 infection in both non-human primates and humans with COVID-19.

### Endothelial Cells Can Be Infected by SARS-CoV-2 *Ex Vivo*

**Figure 4:**
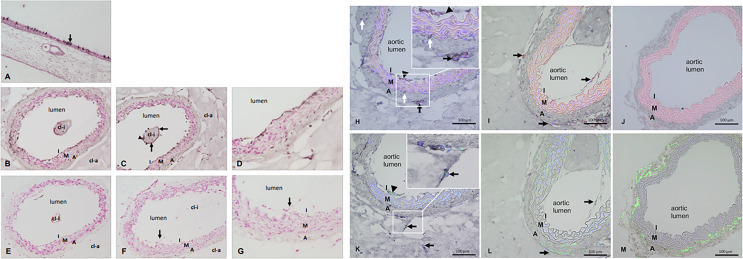
**(A–M)** Immunostaining for SARS-CoV-2 and double immunostainings for SARS-CoV-2 plus ACE-2 or SARS-CoV-2 plus CD34. **(A)** SARS-CoV-2 staining of the nasal mucosa (used as positive control) and single epithelial cells show the dark signals (arrow) indicating SARS-CoV-2 infection. **(B–D)** Tissue sections of mouse aorta after ARA and SARS-CoV-2 infection. Dark signals in the innermost layer called intima (I). Single cells covering the collagen gel area inside the aortic lumen (cl-i) also stained positive for SARS-CoV-2 (arrows) while others are negative (arrowhead). **(E)** ARA section exposed to SARS-CoV-2 infection but treated by secondary antibody only in the immunostaining as control. **(F–G)** Tissue sections of ARA that were not exposed to SARS-CoV-2 infection but stained with both primary and secondary antibodies in the immunostaining procedure. Cells of the aortic intima that sprouted into the aortic lumen are also visible (arrow). Figure panels **(A–G)** Dark staining: SARS-CoV-2 staining, red staining: counterstaining with Calcium red, cl-i: collagen gel inside the aortic lumen, cl-a: collagen gel around the aortic wall, I: intima, M: media, A: adventitia. Figure panels **(H–M)** Double immunostainings for SARS-CoV-2 plus ACE-2 or SARS-CoV-2 plus CD34 on tissue sections of ARA and FIA. **(H)**: Co-localization of SARS-CoV-2 (dark signals) and ACE-2 (red) in intimal ECs and adventitial sprouting cells (arrowhead: intimal ECs; arrows: sprouting adventitial cells). Inset: higher magnification demonstrates the clear co-localization of both dark and red staining signals in the aforementioned cells. Of note, some SARS-CoV-2 positive stained cells (white arrows, dark staining) remained negative for ACE-2. **(I)** only ACE-2 staining (red signals) are detectable in intimal ECs and adventitial sprouting cells (arrows) in ARA without exposure to SARS-CoV-2. **(J)** No specific staining signals are detectable in the tissue section of FIA. Note the autofluorescence signals (red) of elastic laminae of the aortic wall. **(K)** Co-localization of SARS-CoV-2 (dark staining signals) and CD34 (green) in intimal ECs and sprouting adventitial cells (arrowhead: intimal ECs; arrow: sprouting adventitial cells). Inset: higher magnification demonstrates the clear co-localization of both dark and green staining signals in the aforementioned cells. Note that tissue sections in **(H)** and **(K)** were prepared as serial sections from the same ARA, demonstrating a very similar pattern of cellular localization. **(L)** Only CD34 staining (green) is detectable in intimal ECs and sprouting adventitial cells (arrows) in ARA without exposition to SARS-CoV-2. **(M)** Only CD34 staining (green) is detectable in the wall of FIA, particularly strong in cells within the aortic adventitia as expected. I: intima, M: media, A: adventitia.

We further investigated endothelial infection by SARS-CoV-2 in an *ex vivo* organ culture model, the aortic (arterial) ring assay (ARA), which was first established almost 30 years ago (Nicosia and Ottinetti, 1990). ARA is used frequently in angiogenesis research for studying new vessel formation, testing the effect of pro- and anti-angiogenic substances, and examining vascular pathologies (Aplin and Nicosia, 2019). One of the well-known characteristics of this model is the activation of mature endothelial cells to proliferate and migrate into the collagen gel to form capillary-like vascular sprouts while the smooth muscle layer of the arterial wall remains highly stable. The endothelial activation is marked by upregulation of some cell adhesion markers including VCAM-1, ICAM and E-selectin (Qin Z et al., 2021). VCAM-1 has also been reported to be elevated in the blood plasma of COVID-19 patients and was found to be associated with acute vascular inflammation (Jin et al., 2020). Immunostaining for VCAM-1 on tissue sections from ARA and fIA (freshly isolated mouse aorta) demonstrated a clear up-regulation of VCAM-1 in both aortic endothelial cells as well as in capillary-like sprouts that were formed by aortic adventitia-derived endothelial progenitors and sprouted into the collagen matrix around the aortic rings (**Supplemental Figure 6**). The upregulation of VCAM-1 after performing aortic ring assay was also confirmed at the RNA level by conventional and quantitative RT-PCR analyses (**Supplemental Figure 6**). Thus, we considered this assay suitable for testing SARS-CoV-2 endothelial infection since a) it enables the study of endothelial cell behavior in its natural microenvironment, interacting with other cellular and structural components of the aortic wall, and b) it mimics the *in vivo* situation of the vasculature after SARS-CoV-2 infection, where vascular endothelial cells are also activated by infection-related systemic cytokine storm, which modifies endothelial barrier function. To this end, small rings of mouse aorta were prepared and embedded into collagen gel and cultured for 6 days until they started to form sprouts into the collagen gel in both directions (i.e., into the aortic lumen and the collagen gel around the aortic adventitia). The aortic ring was then exposed to SARS-CoV-2 for 3 days. After removing the SARS-CoV-2-rich supernatant, the presence of virus in the aorta was determined by immunohistochemical staining and electron microscopic analyses. SARS-CoV-2-infected human nasal mucosa was used as a positive control and revealed intense SARS-CoV-2 staining in single epithelial cells (**Figure 4A**). The aortic ring sections displayed SARS-CoV-2 immunostaining in the intimal layer (**Figures 4B–D**) and in a few places at the border between the media and the aortic wall’s adventitial layer (**Figure 4B**). Furthermore, some single cells covering the collagen gel matrix area within the aortic lumen were stained positive for SARS-CoV-2 (**Figures 4B, C**). Higher magnification displayed the localization of SARS-CoV-2 staining in endothelial cells lining the aortic lumen (**Figures 4C, D**), while the media layer was almost free of any specific staining. SARS-CoV-2 staining was also observed in new, endothelial lined vascular channels sprouting from the aortic wall, while cells within the matrix were mostly negative (**Figure 4D**). In the tissue section from ARA, which was exposed to SARS-CoV-2 infection as ARA sections in **Figures 4B–D** but treated with secondary antibody only, no specific staining for SARS-CoV-2 could be observed (**Figure 4E**). Similarly, no specific staining was seen in the tissue sections of ARA that were not exposed to SARS-CoV-2 infection but stained with both primary SARS-CoV-2 antibody and the biotinylated secondary antibody (**Figures 4E–G**). These data suggest that ACE2 or another concomitantly upregulated gene product, may mediate SARS-CoV-2 entry into these cells. To further study this matter, we performed double immunostaining on tissue sections of ARA and freshly isolated mouse aorta (FIA) combining DAB-based detection for SARS-CoV-2 (dark signal) and ACE-2 (red immunofluorescence) or CD34 (green immunofluorescence), a marker for mature endothelial and endothelial progenitor cells (**Figures 4H–M**). These data were further confirmed by double immunostaining for VCAM-1 (green immunofluorescence), a marker for endothelial dysfunction and inflammation (Qin Z et al., 2021), and ACE-2 (red immunofluorescence) (**Supplemental Figure 7**). The results from these analyses revealed an upregulation of ACE2 in both activated intimal endothelial cells and sprouting adventitial cells (**Figure 4H, I**) that were also positive for SARS-CoV-2 (**Figures 4H**), while no ACE2 staining could be detected in the wall of the FIA with quiescent endothelial cells (**Figure 4J**). Considering one of the well-known characteristics of ARA is its activation of mature endothelial cell. Our results suggest that activation of the endothelial cells and an upregulation of ACE2 (and perhaps additional factors) sensitize the endothelial cells to SARS-CoV-2 infection. Interestingly, some of the SARS-CoV-2-positive cells remained negative for ACE2 (**Figure 4H**). Results from double immunostaining for SARS-CoV-2 and CD34 (**Figures 4K–L**) and for SARS-CoV-2 and CD31 (**Supplemental Figure 8**) confirmed the endothelial nature of SARS-CoV-2 positive cells of both aortic intima and adventitia (**Figure 4K**), while in ARA sections not exposed to SARS-CoV-2, no specific staining for SARS-CoV-2 was detectable (**Figures 4I, L**), supporting the specificity of the SARS-CoV-2 antibody used in these studies. Finally, in FIA, CD34 positive cells were found in aortic adventitia as published previously (Zengin et al., 2006; Campagnolo et al., 2010; Mekala et al., 2018). Still, as expected, no staining for SARS-CoV-2 was observed, and therefore FIA was used as the negative control (**Figure 4M**).

**Figure 5:**
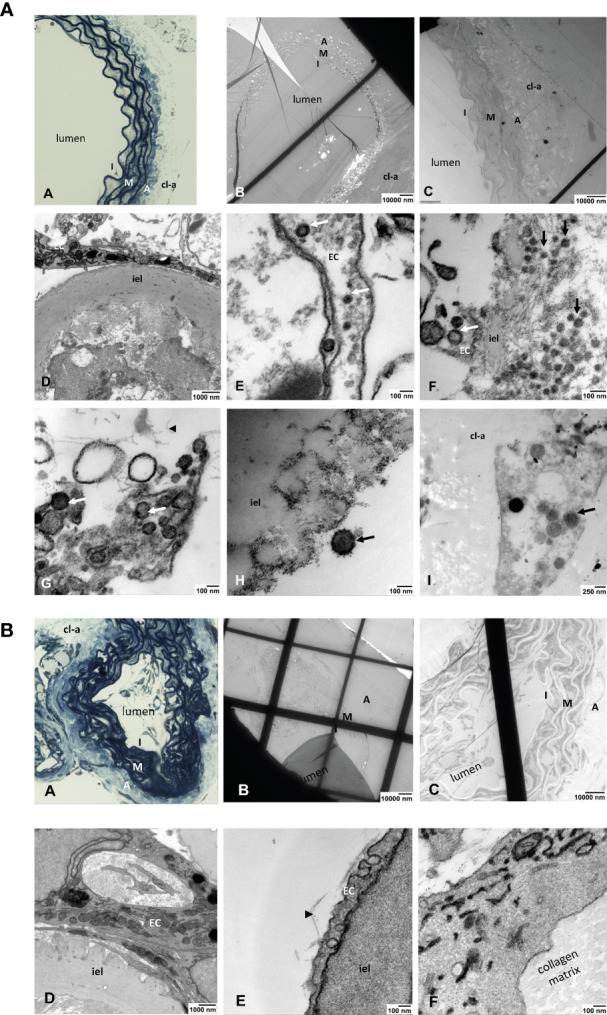
**(A)** Light and electron microscopic analyses of SARS-Cov-2-infected ARA. **(A)** toluidine blue-stained aortic wall section displaying the layers intima (I), media (M) and avdentitia **(A)**. **(B)** Tissue section of SARS-CoV-2-infected ARA in EM with low and **(C)** higher magnification displaying the aortic wall layers as in **(A). (D)** Higher magnification shows the aortic intima with EC and inner elastic lamina (iel) **(E–I)** SARS-CoV-2 viral particles (arrows) in a EC process into the aortic lumen **(E)**, in the intimal layer (arrows) **(F)**, inside of an EC process within the collagen matrix (arrowhead) **(G)**, attached to the inner elastic lamina (iel) **(H)**, in a cell that was migrated into the collagen gel (cl-a) around the aortic wall **(I)**. I: intima, M: media, A: adventitia; EC: endothelial cell; cl-a: collagen gel around the aortic wall; iel: inner elastic lamina of the aortic wall. **(B)** Light and electron microscopic analyses of non-infected ARA. **(A)** Toluidine blue-stained aortic wall section displaying the layers intima (I), media (M) and adventitia **(A)**. **(B, C)** tissue section of non-infected ARA in EM with low and higher magnification displaying the aortic wall layers as in A. **(D–F)**: No SARS-CoV-2 viral particles were detected in the cells of the aortic wall, neither in ECs of the intima **(D, E)** nor in those sprouted into the collagen matrix **(F)**. I: intima, M: media, A: adventitia; cl-a: collagen gel around the aortic wall; iel: inner elastic lamina of the aortic wall.

Electron microscopic studies on ultrathin sections of ARA confirmed the infection of endothelial cells by SARS-CoV-2. We detected viral particles inside the endothelial cells lining the aortic lumen but also in single cells that migrated into the collagen gel outside the aortic wall (**Figure 5A: A–I**). As expected, similar analyses in tissue sections of non-infected ARA used as controls revealed no viral particles (**Figure 5B: A–F**).

### Endothelial and Epithelial Cell Infection Partially Depend on hACE2 Expression

**Figure 6:**
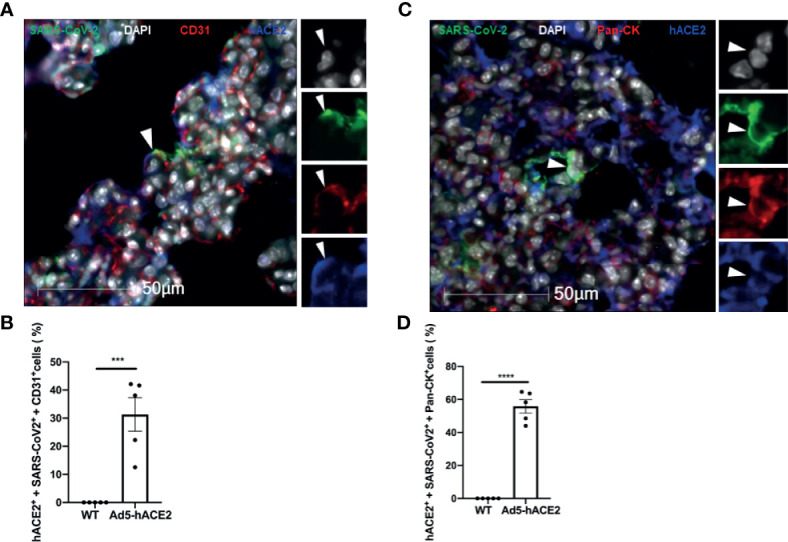
Endothelial cell infection occurs with the presence of hACE2 in the SARS-CoV-2 infected Ad5-hACE2 mice. **(A)** Representative image shows co-localization (arrowhead) of SARS-CoV-2 proteins (green) with hACE2 (blue) and CD31 (red) in the lungs of infected Ad5-hACE2 mice at 3 DPI (n = 5). **(B)** The ratio of infected hACE2^+^ endothelial cells (hACE2^+^, SARS-CoV-2^+^ and CD31^+^) to total infected cells. **(C)** Representative image shows co-localization (arrowhead) of SARS-CoV-2 proteins (green) with hACE2 (blue) and Pan-CK (red) in the lungs of infected Ad5-hACE2 mice at 3 DPI (n = 5). **(D)** The ratio of the infected hACE2^+^ pneumocytes (hACE2^+^, SARS-CoV-2^+^ and Pan-CK^+^) to total infected cells. Multilabel immunofluorescence histochemistry was used to quantify the proportion of SARS-CoV-2 positive cells expressing the markers PanCK and CD31 with or without ACE2, in the infected Ad5-hACE2 mice lung at 3DPI (Mean ± SEM, n = 5, quantitative analysis was performed from 96 and 106 microscopic regions from 5 fluorescent immunohistrochemsitry staining slides of the 5 infected Ad5-hACE2 mice respectively (1 slide per mouse). WT: SARS-CoV-2-infected B6 wild type mice; and Ad5-hACE2: SARS-CoV-2-infected Ad5-hACE2 mice. ***P < 0.001 *vs* WT, and ****P < 0.0001 *vs* WT.

After confirming the infection of endothelial and epithelial cells *in vivo*, we investigated how these cells can be infected by SARS-CoV-2 in the lungs of the Ad5-hACE2 mice. To this end, we quantified the ratio of hACE2 expression in infected endothelial cells and epithelial cells of each infected mouse by co-staining of SARS-CoV-2 and hACE2 with CD31 or Pan-CK. We observed that average 31.31% of the infected endothelial cells (SARS-CoV2+ and CD31+), (**Figures 6A, B**) and average 55.8% of infected epithelial cells (SARS-CoV-2+ and Pan-Ck+), (**Figures 6C, D**) expressed detectable hACE2 in the infected Ad5-hACE2 mice. The hACE2 negative cells among the infected cells could be attributed to the downregulation of hACE2 after the infection and/or the limitation of the immunostaining for detecting low expression of hACE2. This analysis suggests SARS-CoV-2 infects the endothelial cells at least in part through human ACE2.

## Discussion

We demonstrate here that in addition to pulmonary epithelial cells, vascular endothelial cells, including endothelial cells of pulmonary vessels, are also vulnerable to SARS-CoV-2 infection, which may contribute to the pathogenesis of COVID-19 and worsen the clinical outcome. SARS-CoV-2 infection of ECs is only seen in septa, which carry out the critical function of gas exchange across the pneumocyte-endothelial barrier. This could explain, at least partially, the shortness of breath and low O_2_ saturation in COVID-19 patients: Increased pulmonary vascular permeability and inflammation due to endothelial infection in the area may cause edema, hemorrhage, and microvessel thrombosis, affecting gas exchange in the infected lung. Previous clinical studies using immune histological and electron microscopic methods showed the presence of SARS-CoV-2 within the endothelial cells of lungs and other organs and an accumulation of inflammatory cells as well as endothelial and inflammatory cell death (Ackermann et al., 2020; Colmenero et al., 2020; Fox et al., 2020; Varga et al., 2020a). These histological findings of vascular damage and endothelial infection support a causal relationship between this damage with SARS-CoV-2 infection (Ackermann et al., 2020; Colmenero et al., 2020; Fox et al., 2020; Varga et al., 2020a). However, whether the infected endothelial cell results seen in humans are false positives is still a matter of debate (Goldsmith et al., 2020; Varga et al., 2020a; Varga et al., 2020b). In our study, we used multiple tools including immunostaining on tissue sections, RNAscope, and conventional immune electron microscopic analysis to examine endothelial cell infection in the lungs of our previously established COVID-19 mouse and NHP models at both the early (3 DPI for the mouse and 4-6 DPI for NHPs) and the later course of the disease. It is important to note that detecting infected endothelial cells in the lungs of the mouse and NHP models is much easier than for patients who died from COVID-19. From three COVID-19 autopsy cases used, we could only detect a few infected intact endothelial cells in two cases. This can be attributed to the peak SARS-CoV-2 load observed in the early course but not in the later course of the diseases, as also observed in our mouse (Han et al., 2021) and NHP models. This is supported by clinical observations showing the highest viral load at the time of symptom onset or in the first week of illness, with subsequent decline thereafter, indicating the highest infectiousness potential just before or within the first five days of symptom onset (Cevik et al., 2020). Autopsy studies of lungs of COVID-19 patients who developed respiratory failure showed that SARS-CoV-2 infection of epithelial cells could easily be detected during the acute phase of lung injury but not in the late phase (Schaefer et al., 2020). Using *in situ*-hybridization of SARS-CoV-2 RNA analysis of multiple tissues of 32 confirmed cases, a recent study found only two case-patients with endothelial infection (Bhatnagar et al., 2021). One case had endothelial cell infection in multiple organs and one with had endothelial cell infection in the alveolar capillary (Bhatnagar et al., 2021).

In addition, through mouse aorta ring assay (ARA), we documented that SARS-CoV-2 infects endothelial cells in this organoid culture system that mimics, to some extent, the disease-related activation of vascular endothelial cells and subsequent cytokine storm, which might make endothelial cells more susceptible to SARS-CoV-2. Indeed, our present results obtained from ARA show an upregulation of ACE2 in such activated endothelial cells that were also positive for SARS-CoV-2 staining. In contrast, quiescent endothelial cells of the freshly isolated aorta (FIA) remained negative for ACE2. Consistently, SARS-CoV-2 upregulates the expression of a subset of KRAS-responsive genes involved in endothelial activation and angiogenesis. Interestingly, activation of KRAS signaling pathways in the vascular endothelium has also been recognized to induce brain vascular malformations and hemorrhagic stroke (Li et al., 2018).

Our results are consistent with previous findings showing that engineered human capillary organoids generated from induced pluripotent stem cells can be infected with SARS-CoV-2 (Monteil et al., 2020). However, recent studies using either endothelial cell lines or primary endothelial cells from different human organs document that endothelial cells are resistant to SARS-CoV-2 infection due to low or no expression of ACE2 (Conde et al., 2020; McCracken et al., 2021). These distinct responses to SARS-CoV-2 infection between the endothelial cells of ARA or in organoid culture system and the cell culture system may be explained by the fact that cultured endothelial cells are probably not activated enough to express ACE2 or they do not have the structural support and cellular communication with other cell types that make up the vessel wall structure. In intact vessels, endothelial cells are in direct or indirect contact with epithelial cells, pericytes, smooth muscle cells, endothelial progenitor cells, and possibly other tissue-resident cells, but these interactions are absent in endothelial cell monocultures. This prediction is supported by the recent finding that endothelial cells are infected by SARS-CoV-2 when they are co-cultured with alveolar epithelial cells (Wang et al., 2020). Thus, these data, along with the results obtained in the organoid cultures, support the idea that infection of ECs by SARS-CoV-2 may require unidentified signaling events or activation because it must occur in the context of vascular structure and in the presence of other cell types.

Our results indicate that endothelial cells can be directly infected by SARS-CoV-2 *in vivo* and *in vitro*. However, while our data obtained from ARA studies suggest upregulation of ACE2 upon endothelial activation as a possible manner of SARS-CoV-2 entry into endothelial cells, the underlying mechanism by which SARS-CoV-2 infects endothelial cells *in vivo* remains unclear. In addition, ACE2 is reported to be expressed by endothelial cells in organs including lung, heart, kidney, and intestine (Ferrario et al., 2005), and under quiescent conditions, the ratio is only 1/250 (Ackermann et al., 2020) or even lower as compared to epithelial cells (Ferrario et al., 2005; Soler et al., 2009; Ziegler et al., 2020). However, whether the infection requires or uses an additional viral entry receptor is unknown and needs further investigation. Recent studies have shown that alternative receptors or host factors could be involved in the entry of SARS-CoV-2 into host cells (Cantuti-Castelvetri et al., 2020; Daly et al., 2020; Ganier et al., 2020; Ichimura et al., 2020; Wang et al., 2020; Shilts et al., 2021; Wang et al., 2021). For example, CD147, a transmembrane glycoprotein of the immunoglobulin superfamily, has been identified as a potential receptor to bind spike protein and mediate virus entry (Wang et al., 2020). Interestingly, single-cell RNA sequencing data also demonstrates that CD147, but not ACE2, is detectable in human vascular endothelial cells (Ganier et al., 2020), indicating that CD147 may be a potential receptor for endothelial infection. However, a recent *in vitro* study did not find evidence of direct binding of CD147 to the spike protein of SARS-CoV-2 (Shilts et al., 2021), which warrants further study to clarify the interaction between them, particularly in endothelial cells. Another interesting host factor that could increase SARS-CoV-2 infectivity is neurophilin-1 (NRP1). Two independent studies demonstrate that the cell surface receptor NRP1, which is known to bind furin-cleaved substrates, binds directly to the maturated fragments of spike protein and facilitates entry of SARS-CoV-2 into cells with low ACE-2 expression. These results suggest NRP1 could serve as a find-me signal to direct SARS-CoV-2 to the target cells (Cantuti-Castelvetri et al., 2020; Daly et al., 2020). Of note, although ACE2 expression is low in pulmonary epithelial cells and ECs, NRP1 is abundantly expressed in these cells, indicating NRP1 may be a necessary host factor for efficient infection and spreading. Like many viruses, SARS-CoV-2 may use multiple receptors/host factors, including hACE2-dependent and independent mechanisms to enter host cells. Given the fact that there is low ACE2 expression in quiescent ECs (McCracken et al., 2021) and our data that SARS-CoV-2 infect ECs *in vivo* and *in vitro*, it will be interesting to investigate potential co-receptor(s) or host factors in ECs in a future study.

In summary, we demonstrate that SARS-CoV-2 can infect vascular endothelial cells, including pulmonary endothelial cells *in vivo* and *in vitro* an organ culture system. These results highlight the importance of ECs in COVID-19 pathogenesis and prevention and treatment of endothelial infection and injury in COVID-19 patients, especially for high-risk patients with chronic cardiovascular diseases.

## Data Availability Statement

The raw data supporting the conclusions of this article will be made available by the authors, without undue reservation.

## Ethics Statement

The animal study was reviewed and approved by Tulane University. Written informed consent was obtained from the individual(s), and minor(s)’ legal guardian/next of kin, for the publication of any potentially identifiable images or data included in this article.

## Author Contributions 

FL, KH, RB, ZQ, KK, JK, JR, SE, and XQ developed the concept. FL, KH, RB, KK, BU, JB, LD, PW, ZQ, CM, JM, TR, NM, EB, JK, RV, X-MY, JR, SE, and XQ contributed to perform the experiments and analyzed the results. RSH, TR, NM, EB, RSVH, X-MY, and CR provided the nonhuman primates and human samples. FL, KH, RB, ZQ, KK, BA, JK, RV, XM, CR, JR, SE, and XQ wrote the manuscript, and all authors participated in the review and critique of the manuscript. SE and XQ interpreted the results and supervised the experiments. All authors contributed to the article and approved the submitted version.

## Funding

This work was supported by NIH 5 P51OD011104-58 (JR), R21OD024931 (XQ), 1R21AI154196 (BHA), Gates foundation (XQ and JR), Tulane start-up funds (XQ, and JP), and Tulane COVID-19 biobank (EB). The following reagent was deposited by the Centers for Disease Control and Prevention and obtained through BEI Resources, NIAID, NIH: SARS-Related Coronavirus 2, Isolate USA-WA1/2020, NR-52281. This research was funded by the Deutsche Forschungsgemeinschaft (DFG, German ResearchFoundation)—Projektnummer 374031971—TRR 240 to SE & HS (A03) and DS (B06).

## Conflict of Interest

The authors declare that the research was conducted in the absence of any commercial or financial relationships that could be construed as a potential conflict of interest.
