# Supplementary material for: SARS-CoV-2 Infects Endothelial Cells In Vivo and In Vitro
Source: Front Cell Infect Microbiol. 2021 Jul 6;11:701278. doi: 10.3389/fcimb.2021.701278 (PMC8292147; doi:10.3389/fcimb.2021.701278)
Supplement: Supplementary file 1 [file DataSheet_1.pdf]

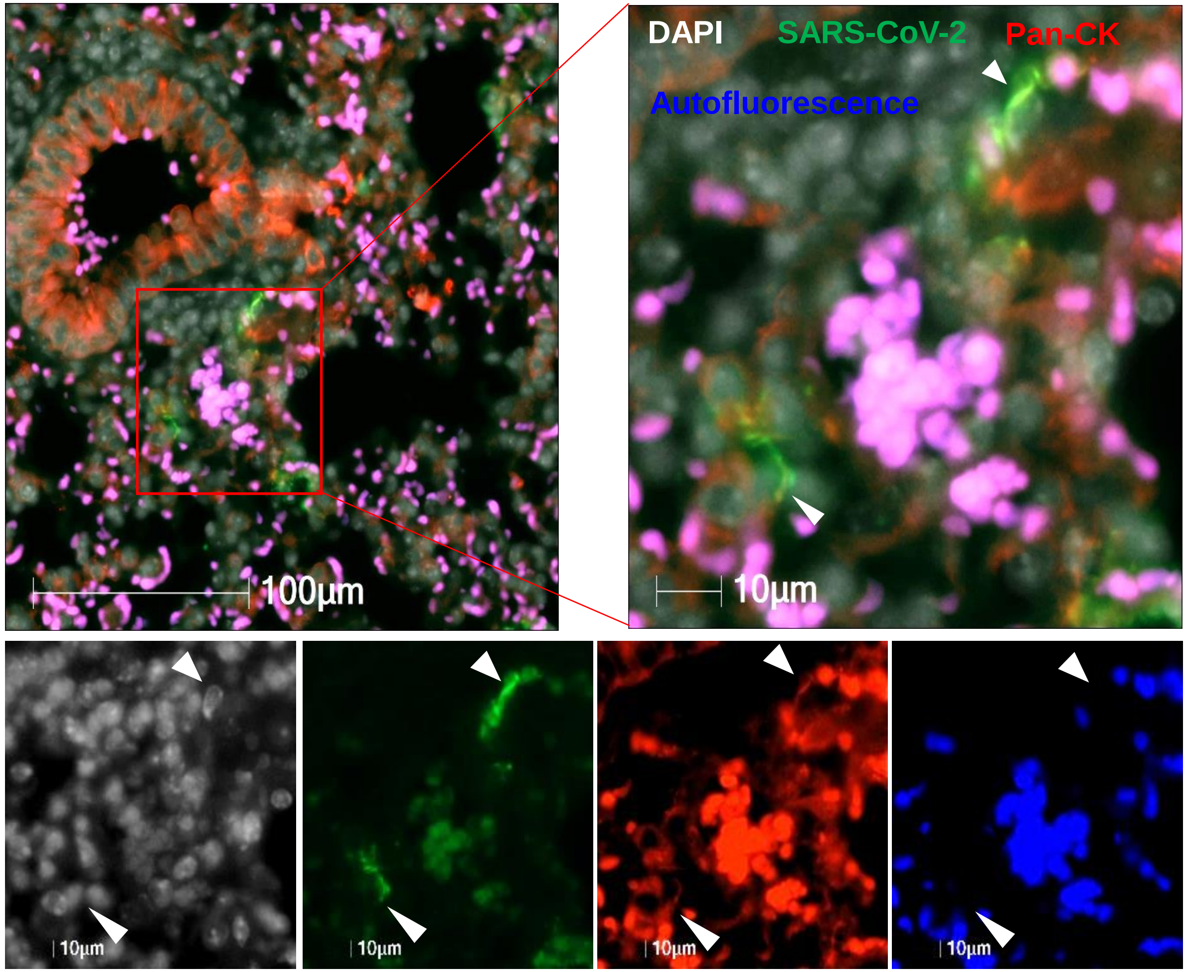

Supplemental Figure 1: SARS-CoV-2 infection of pneumocytes in Ad5-hACE2 mice lungs. Co-localization (arrowhead) of SARS-CoV-2 proteins (green) with Pan-CK in (red) infected Ad5-hACE2 mice lungs on 3DPI (n = 5). Green: SARS-CoV-2; white: DAPI; red: Pan-CK; blue: autofluorescence.

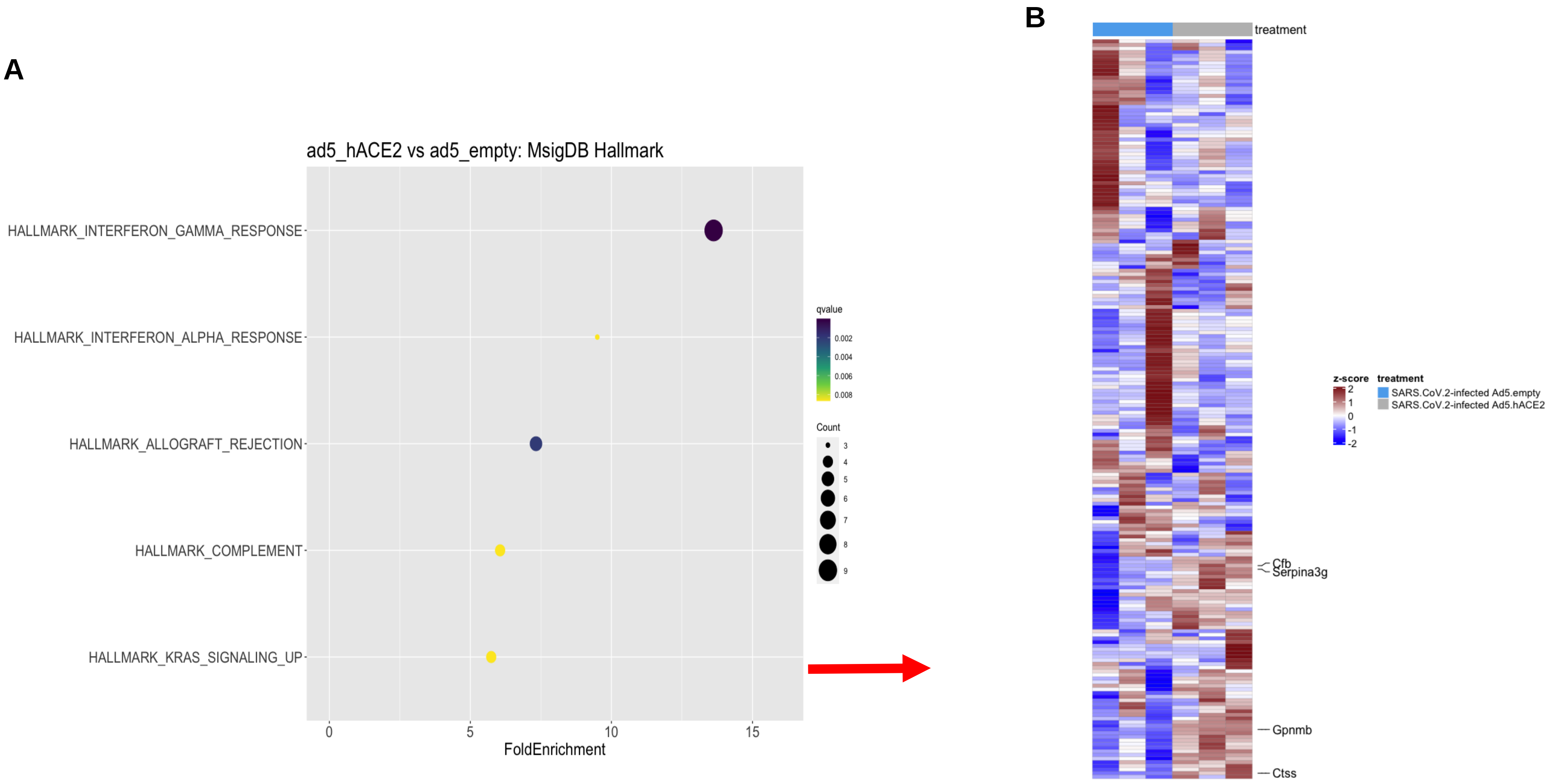

Supplemental Figure 2. Biological pathways related to K-ras signaling are enriched the lungs of Ad5-hACE2 mice (n = 3) as compared to Ad5-empty (n = 3) upon SARS-CoV-2 infection. (**A**). Fold enrichment of top pathways within the MsigDB collection, "hallmark" gene set. Color ramp represents fold enrichment defined by a Fisher's Exact test. Dot size represents 'setSize', or number of genes contained within an *a priori*-defined biological pathway. (**B**) Composite heatmap of genes within the MsigDB "HALLMARK_KRAS_SIGNALING_UP" gene set. Genes differentially expressed in this pathway are annotated. Rows are scaled by z-score-normalized expression.

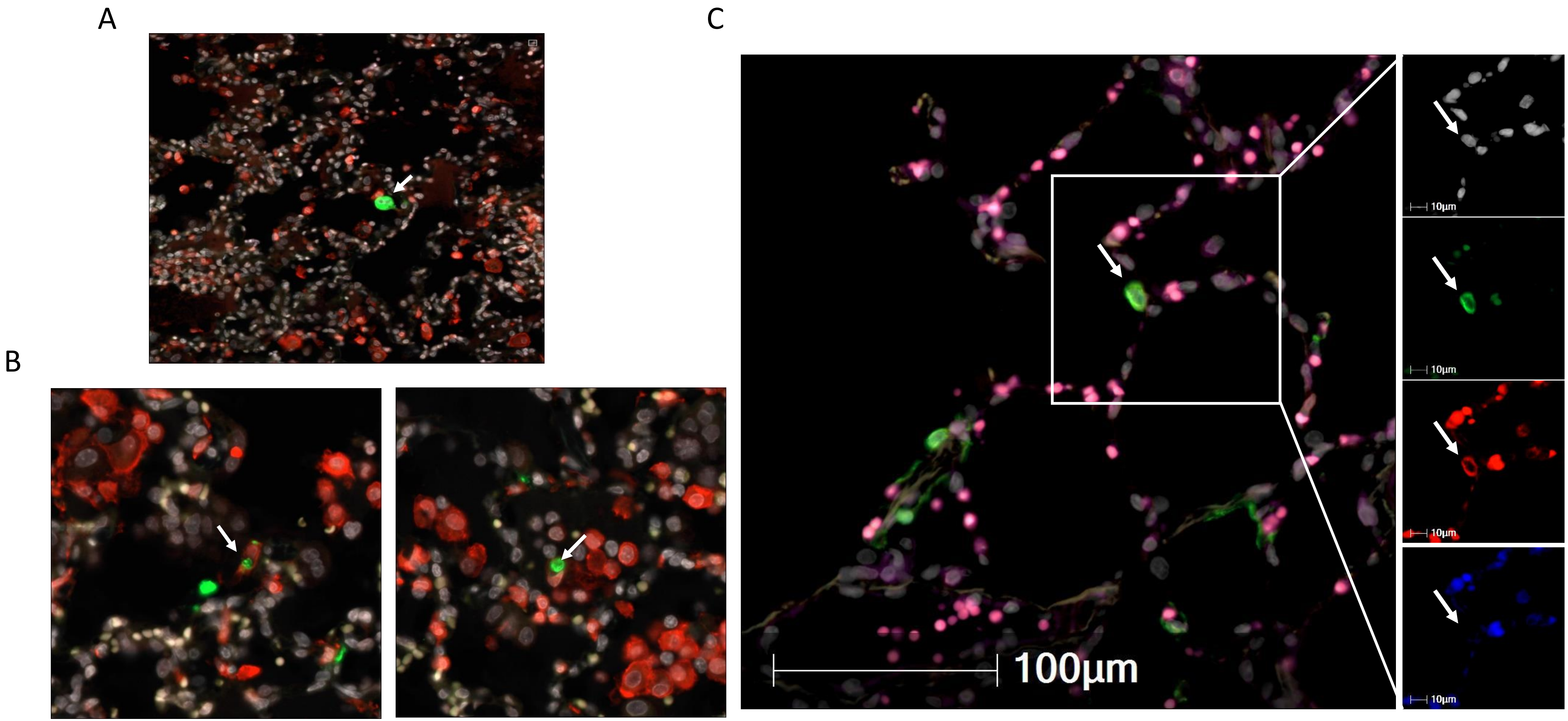

Supplemental Figure 3: Representative **SARS-CoV-2 staining in macrophage and T cells in the lung of infected non-human primates.** (**A**) SARS-CoV-2 antigen (green) does not appear to colocalize with the macrophage marker IBA-1 (red) (n = 3). Even in cells with similar morphology to alveolar macrophages (arrow); (**B**) Macrophages rarely contain phagocytosed debris from SARS-CoV-2 infected cells (green, arrows) (n = 3). However, the localized distribution within the cells suggests the macrophages (red) is not infected. (**C**) Arrow indicates the co-localization of SARS-CoV-2 (Green) and CD3 (Red) (n = 3).

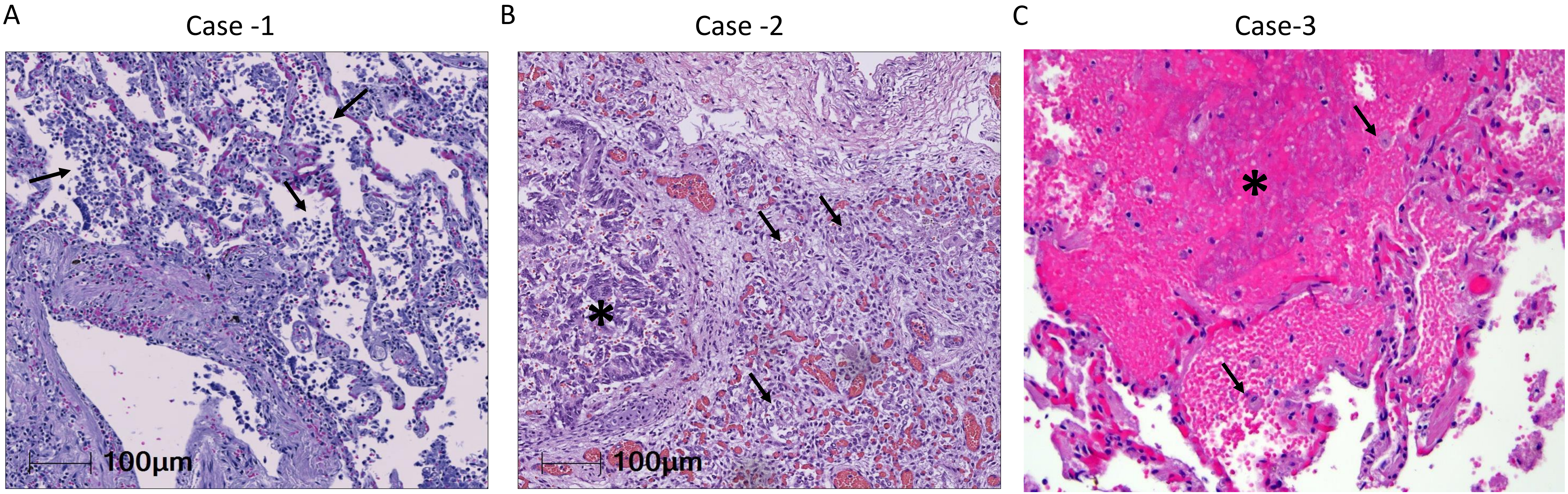

Supplemental Figure 4: H & E staining of the lungs of the cases 1, 2 and 3 COVID-19 patients. (**A**) Autopsy lung with marked acute and chronic inflammatory cells in the intra-alveolar space (arrows). (**B**) Fetal autopsy lung showing immature pulmonary tissue with a bronchus adjacent to developing alveoli (asterisks). Intra-alveolar fibrin deposition is present. (**C**) Autopsy lung with diffuse hemorrhage (asterisks) and occasional pigmented macrophages (arrows).

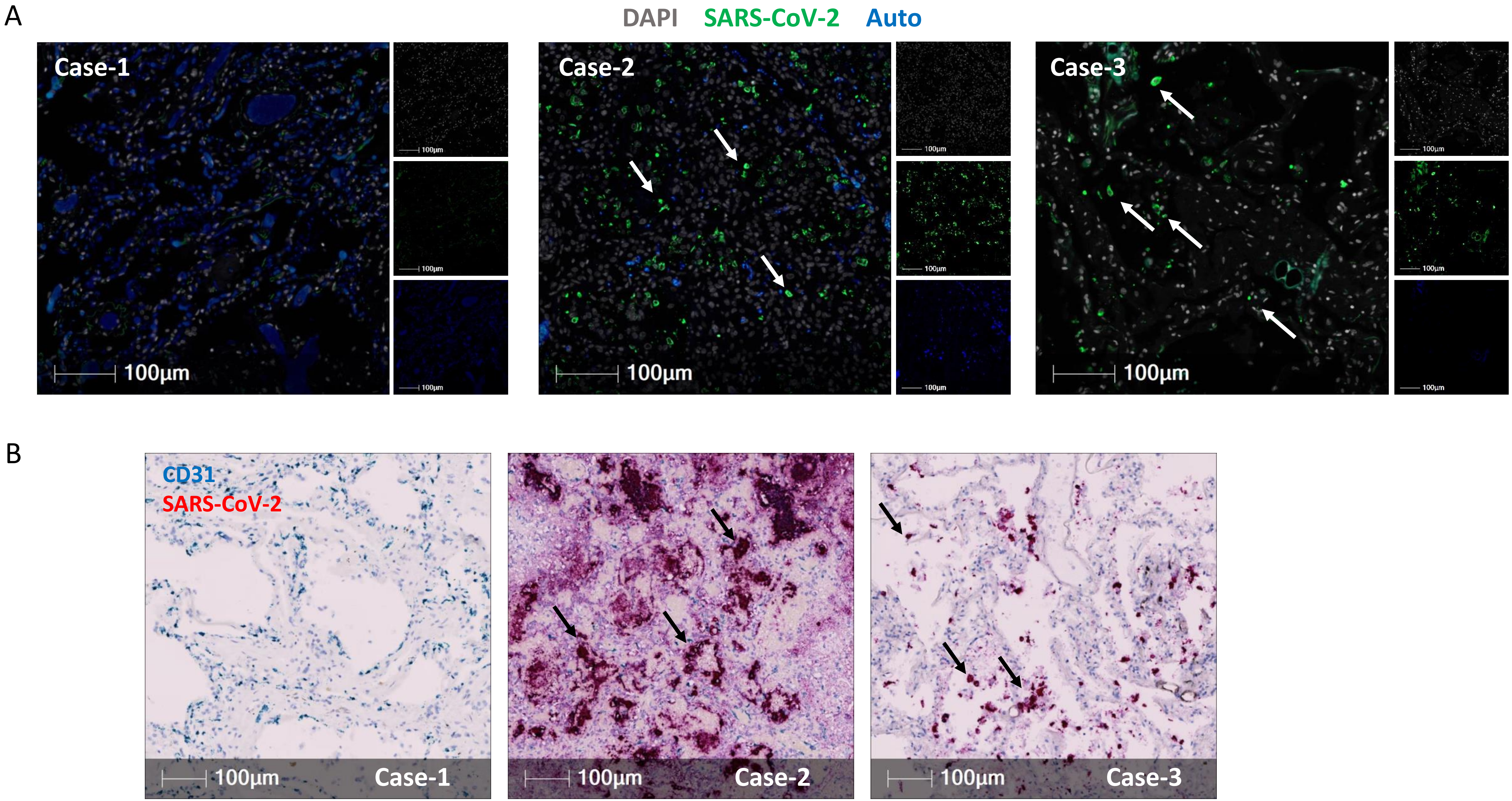

Supplemental Figure 5: Representative IHC staining (A) and RNAscope staining (B) of the lung sections from 3 COVID-19 patients. SARS-CoV-2 (arrows) was not detected in Case-1, was abundant in Case-2, and multifocal in Case-3.

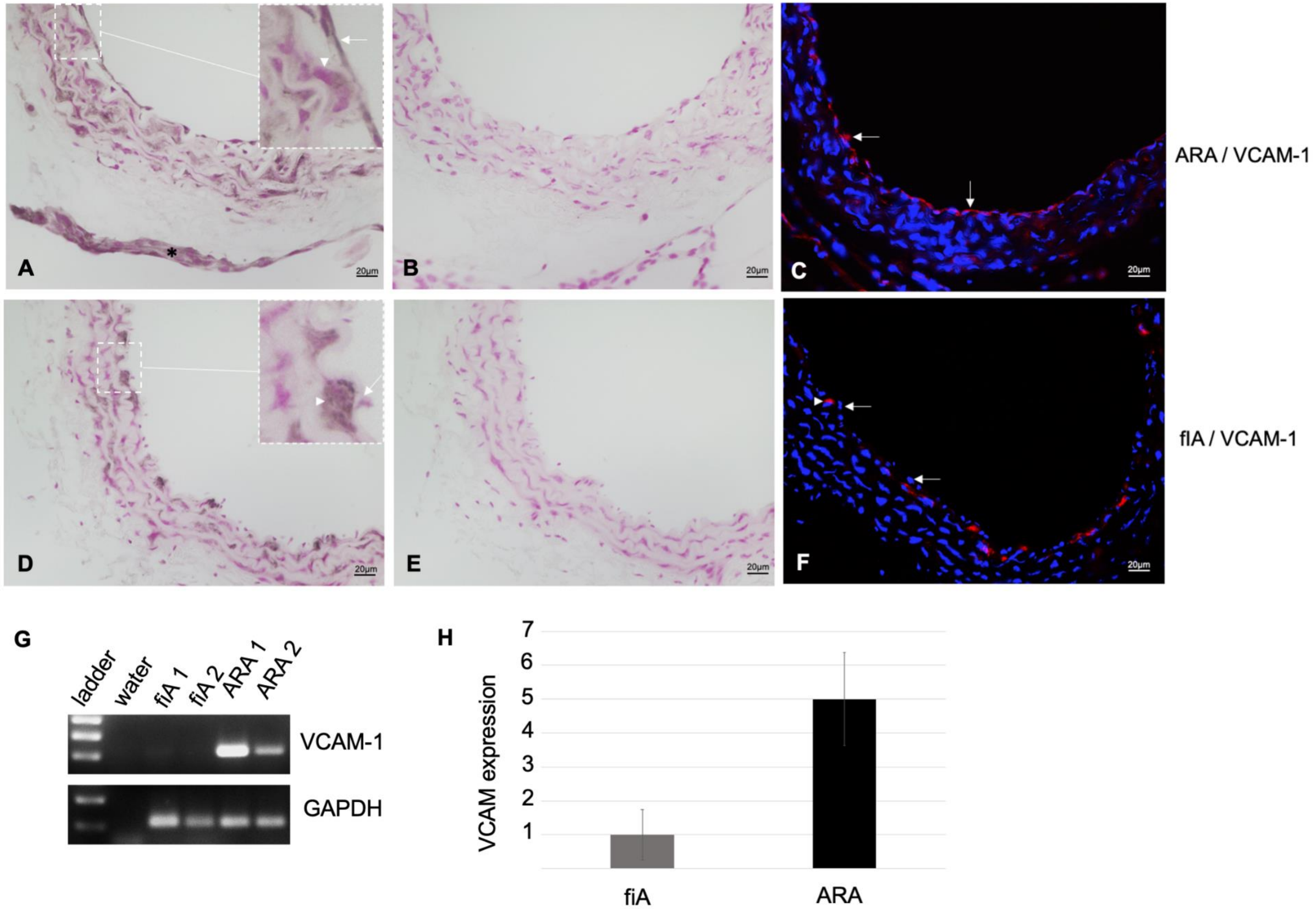

Supplemental Figure 6: Upregulation of VCAM-1 during aortic ring assay (ARA). A: VCAM-1 expression (dark staining) in the aortic endothelium (white arrow) and in vascular sprouts (black asterisk) during aortic ring assay detected using specific VCAM-1 antibody and DAB staining. White arrowhead marks a subendothelial cell. **B:** Secondary antibody control for **A**. **C:** Immunofluorescence analysis detecting the expression of VCAM-1 (red) in the aortic endothelium (white arrow) after ring assay. **D:** VCAM-1 expression is almost absent in the endothelium of freshly isolated aortic rings (white arrow) but can be detected in few subendothelial cells (white arrowhead). **E:** Secondary antibody control for **D**. **F:** Immunofluorescence analysis showing no expression of VCAM-1 in the aortic endothelium (white arrow) in freshly prepared aortic rings. However, VCAM-1 expression (red) is detectable in few subendothelial cells (white arrowhead). **G**: Semiquantitative RT-PCR analyses detecting the expression of VCAM-1 and GAPDH mRNA in 2 samples of freshly isolated aorta (fiA1 and fiA2) and 2 samples collected after aortic ring assay (ARA1 and ARA2). **H**: Quantitative RT-PCR analyses detecting the expression of VCAM-1 and GAPDH mRNA in freshly isolated aorta (fiA) and samples prepared after aortic ring assay (ARA) (n=2).

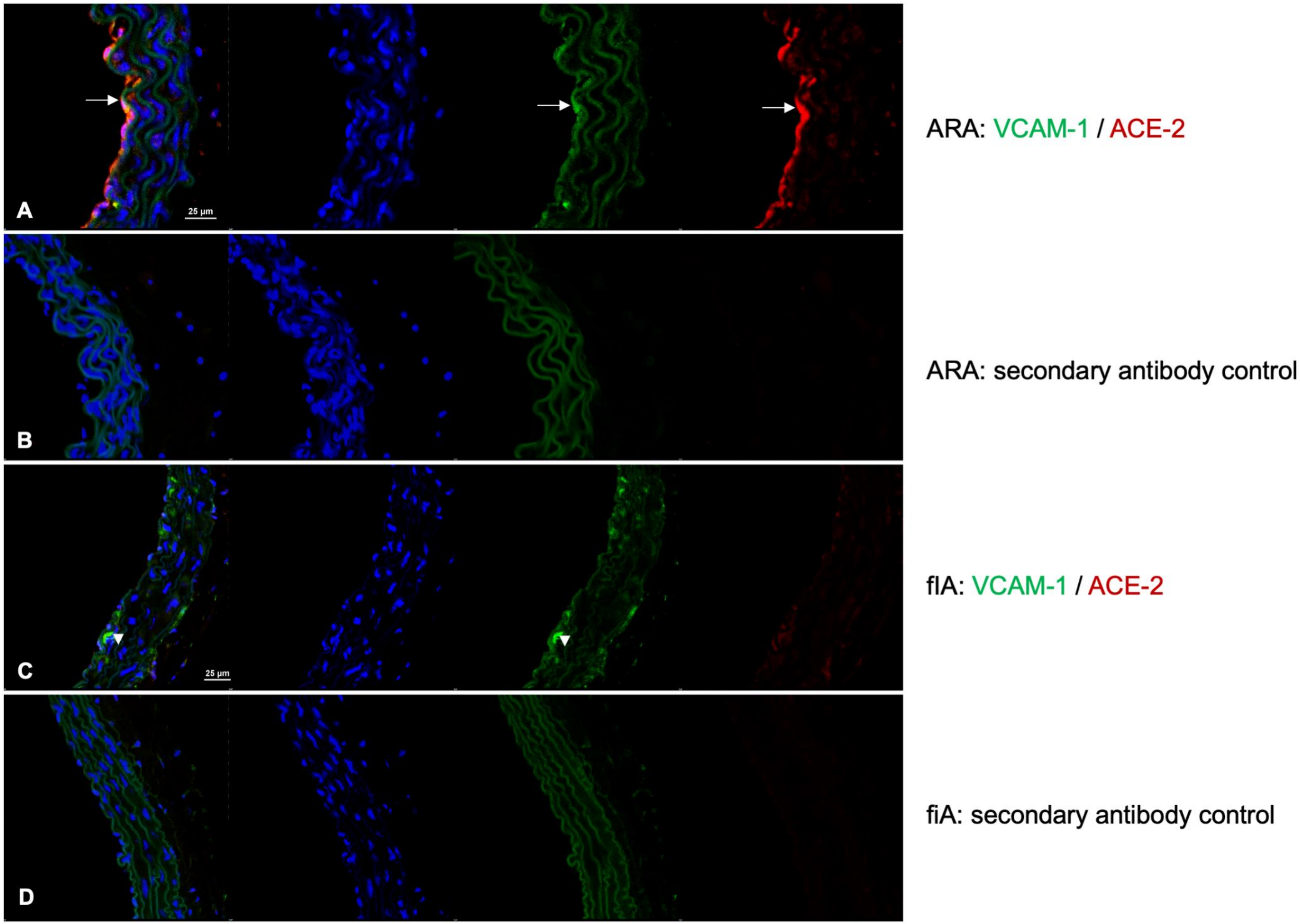

Supplemental Figure 7: Simultaneous upregulation of ACE-2 and VCAM-1 during aortic ring assay (ARA). **A-B**: Immunofluorescence analyses detecting VCAM-1 (green) and ACE-2 (red) protein expression in sections prepared after aortic ring assay (ARA) and **C-D**: from freshly isolated aortic tissue. B and D show secondary antibody controls. The white arrow points towards the aortic endothelium and the white arrowhead marks a VCAM-1^{+} subendothelial cell.

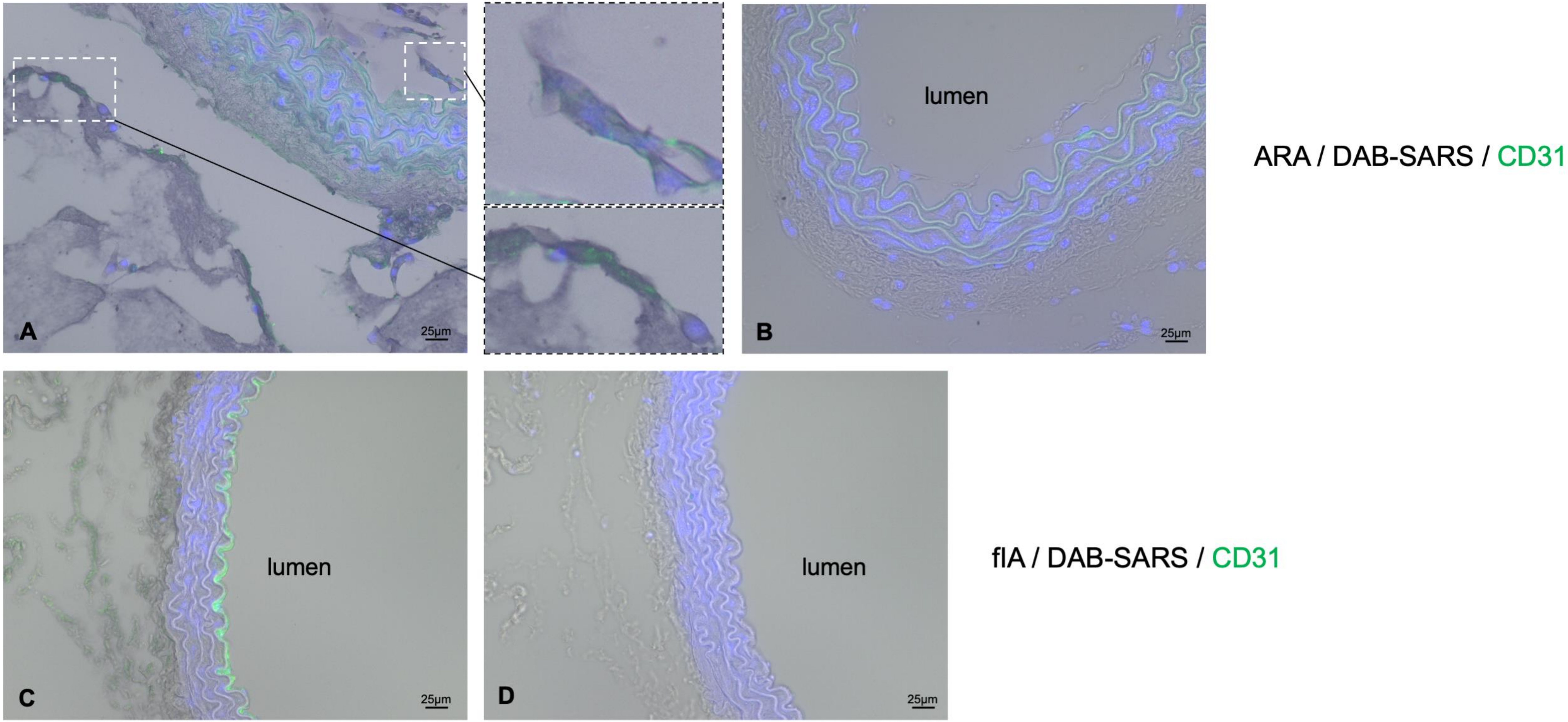

Supplemental Figure 8: Detection of SARS-Cov-2 in the activated aortic endothelium and in sprouting endothelial cells during aortic ring assay (ARA)

A: SARS-Cov-2 (dark staining) is detected in $CD31^+$ endothelial sprouts after ARA using combined DAB staining for SARS-Cov-2 (dark) and immunofluorescence analyses for CD31 (green). The insets highlight endothelial sprouts into the aortic lumen (upper panel) and into the collagen matrix surrounding the rings (lower panel) that demonstrate the co-localization of CD31 and SARS-Cov-2. **B**: Secondary antibody control for A. **C**: No SARS-Cov-2 staining (dark DAB signal) is detected in the $CD31^+$ aortic endothelium (green immunofluorescence) in sections prepared from freshly isolated aortic tissue. **D**: Secondary antibody control for C.
