# Supplementary material for: SARS-CoV-2 Infects Endothelial Cells In Vivo and In Vitro
Source: Front Cell Infect Microbiol. 2021 Jul 6;11:701278. doi: 10.3389/fcimb.2021.701278 (PMC8292147; doi:10.3389/fcimb.2021.701278)
Supplement: Supplementary file 2 [file DataSheet_2.pdf]

Supplemental materials:

Case 1 (TMC-20-1)

The patient is a 58-year-old male with past medical history significant for hypertension, type 2 diabetes mellitus, hepatitis C virus and chronic kidney disease, who was admitted for COVID-19 pneumonia. One week into his hospitalization, it was complicated by hypoxic respiratory failure requiring endotracheal intubation, end-stage renal disease (ESRD) requiring dialysis, and anoxic brain injury secondary to hypotension and shock. Following these complications, he remained persistently unresponsive, and a tracheostomy was performed. At just over a month into his hospitalization, the patient rapidly desaturated, developing hypoxemia, bradycardia, and death due to asystole. Autopsy findings of the lungs are notable for extensive acute bronchopneumonia with focal colonies of cocci bacteria. The lungs also showed multifocal organizing pneumonia with predominantly fibrosing and focally fibrinous features, which were possibly related to background COVID-19 infection (**Supplemental Figure 4A**). Additional notable findings include centrilobular and midzonal necrosis of the liver consistent with severe ischemic necrosis, and the kidneys showed extensive interstitial fibrosis, interstitial inflammation, and glomerulosclerosis consistent with his history of chronic kidney disease. Other findings include hypertrophic cardiomegaly, severe calcific atherosclerosis of the coronary arteries, splenomegaly with congestion of red pulp, and diffuse reactive lymphadenopathy.

Case 2 (TMC-20-2)

The patient is a 4-day-old male neonate delivered prematurely by Cesarean section at 25.6 weeks gestational age due to the pregnancy complications including chronic hypertension and preeclampsia with severe features. The mother tested positive for SARS-CoV-2 on the date of admission upon routine screening, but was clinically asymptomatic and without known exposure. The patient required endotracheal intubation soon after delivery. He tested positive for SARS-CoV-2 at both 24 and 72 hours of life. On day 3 of hospitalization, he demonstrated increased oxygen requirements and hypotension, which decompensated, leading to his death on 10/09/2020. The autopsy revealed diffuse alveolar damage (DAD) (**Supplemental Figure 4B**) and necrotizing fungal pneumonia. Growth parameters and size of internal organs were consistent with normal for gestational age. No congenital malformations were identified.

Case 3: The patient was a 78-year-old African American female with a history of end-stage renal disease, type 2 diabetes and obesity (BMI 34.3) who presented in cardiac arrest after a two-day history of shortness of breath. She was intubated and died the next day. Her lungs exhibited early changes of COVID-19 disease and thrombosis (**Supplemental figure 4C**). These included focal hemorrhage and severe alveolar edema with early hyaline membrane formation. She had numerous clots in the pulmonary vessels and severely elevated d-dimer levels (10,020 ng/ml; normal <250 ng/ml). The edema and hyaline membranes are early changes associated with diffuse alveolar damage.
