# Supplementary material for: SARS-CoV-2 Infects Endothelial Cells In Vivo and In Vitro
Source: Front Cell Infect Microbiol. 2021 Jul 6;11:701278. doi: 10.3389/fcimb.2021.701278 (PMC8292147; doi:10.3389/fcimb.2021.701278)
Supplement: Supplementary file 3 [file DataSheet_3.pdf]

Supplemental Table 1. Animal information for nonhuman primates.

Id	Species	Age (Years)	Sex	Route	Days Post Infection
PA24	GREEN/VERVET	19.98	MALE	IN/IT	4
PA20	GREEN/VERVET	20.10	FEMALE	IN/IT	6
PA16	GREEN/VERVET	19.01	MALE	IN/IT	4

IN intranasal
IT intratracheal
